# From Inflammation to the Onset of Fibrosis through A_2A_ Receptors in Kidneys from Deceased Donors

**DOI:** 10.3390/ijms21228826

**Published:** 2020-11-21

**Authors:** Elena Guillén-Gómez, Irene Silva, Núria Serra, Francisco Caballero, Jesús Leal, Alberto Breda, Rody San Martín, Marçal Pastor-Anglada, José A. Ballarín, Lluís Guirado, Montserrat M. Díaz-Encarnación

**Affiliations:** 1 Molecular Biology Laboratory, Fundació Puigvert, 08025 Barcelona, Spain; 2 Nephrology Department, Fundació Puigvert, 08025 Barcelona, Spain; isilva@fundacio-puigvert.es (I.S.); nserra@fundacio-puigvert.es (N.S.); jballarin@fundacio-puigvert.es (J.A.B.); lguirado@fundacio-puigvert.es (L.G.); 3 Institut Investigació Biosanitaria Sant Pau, Fundación Renal Iñigo Álvarez de Toledo (FRIAT), REDinREN, Autonomous University of Barcelona (UAB), 08025 Barcelona, Spain; 4 Renal Transplant Unit, Fundació Puigvert, 08025 Barcelona, Spain; 5 Department of Emergency Medicine and Transplant Coordination, Hospital de la Santa Creu i Sant Pau, Universitat Autònoma de Barcelona, 08041 Barcelona, Spain; FCaballero@santpau.cat (F.C.); jleal@santpau.cat (J.L.); 6 Urology Department, Autonomous University of Barcelona (UAB), Fundació Puigvert, 08025 Barcelona, Spain; abreda@fundacio-puigvert.es; 7 Molecular Pathology Laboratory, Institute of Biochemistry and Microbiology, Faculty of Sciences, Universidad Austral de Chile, 5110566 Valdivia, Chile; rodysanmartin@uach.cl; 8 Department of Biochemistry and Molecular Biomedicine, Institute of Biomedicine (IBUB), University of Barcelona, National Biomedical Research Institute of Liver and Gastrointestinal Diseases (CIBER EHD), 08028 Barcelona, Spain; mpastor@ub.edu; 9 Institut de Recerca Sant Joan de Déu (IR SJD), 08950 Esplugues de Llobregat Barcelona, Spain

**Keywords:** fibrosis, macrophage, inflammation, transplant, kidney, purinome, adenosine receptor

## Abstract

Pretransplant graft inflammation could be involved in the worse prognosis of deceased donor (DD) kidney transplants. A2A adenosine receptor (A_2A_R) can stimulate anti-inflammatory M2 macrophages, leading to fibrosis if injury and inflammation persist. Pre-implantation biopsies of kidney donors (47 DD and 21 living donors (LD)) were used to analyze expression levels and activated intracellular pathways related to inflammatory and pro-fibrotic processes. A_2A_R expression and PKA pathway were enhanced in DD kidneys. A_2A_R gene expression correlated with TGF-β1 and other profibrotic markers, as well as CD163, C/EBPβ, and Col1A1, which are highly expressed in DD kidneys. TNF-α mRNA levels correlated with profibrotic and anti-inflammatory factors such as TGF-β1 and A_2A_R. Experiments with THP-1 cells point to the involvement of the TNF-α/NF-κB pathway in the up-regulation of A_2A_R, which induces the M2 phenotype increasing CD163 and TGF-β1 expression. In DD kidneys, the TNF-α/NF-κB pathway could be involved in the increase of A_2A_R expression, which would activate the PKA–CREB axis, inducing the macrophage M2 phenotype, TGF-β1 production, and ultimately, fibrosis. Thus, in inflamed DD kidneys, an increase in A_2A_R expression is associated with the onset of fibrosis, which may contribute to graft dysfunction and prognostic differences between DD and LD transplants.

## 1. Introduction

Many factors specific to kidney transplant recipients (KTR) and donors influence the outcome of kidney transplantation [1,2,3] and prognostic differences between living and deceased grafts are well known. Despite this, little is known about the basal inflammatory status of donors at the time of donation [4]. Immunological activation during brain death results in infiltration of leukocytes, macrophages, and dendritic cells prior to kidney procurement [5,6,7], which can damage kidneys [8] and impact on short- and long-term renal graft function [9,10]. We showed previously that circulating monocytes from KTR are associated with prognosis in kidney transplantation [11]. Furthermore, different studies indicated macrophages accumulation in allograft biopsies of KTR [12], which is associated with renal interstitial fibrosis and tubular atrophy and graft outcome [13]. In fact, our group demonstrated that macrophage infiltration into renal grafts of deceased donors before transplantation is associated with long-term renal function [14]. Pre-implantation renal biopsies from deceased donors showed greater inflammation than those from living donors and this appeared to be mediated by M1 macrophages, although the presence of M2 macrophages was also observed, suggesting the coexistence of both phenotypes and even intermediate phenotypes [14]. Macrophage phenotype is modulated by adenosine, diminishing M1 proinflammatory macrophage activation and polarizing macrophages to an M2 anti-inflammatory phenotype [15,16]. Extracellular adenosine produced from hydrolysis of ATP (primarily by the ectoenzymes CD39 and CD73) mediates its effects via activation of G-protein-coupled receptors (A1, A2A, A2B, and A3). In the peritransplant period or during ischemia/reperfusion, ATP is released early after cell damage/death, inducing activation of immune cells that migrate to the site of injury [17,18]. Pannexin (Panx)-1 forms transmembrane channels that release ATP into the extracellular space. It is expressed ubiquitously and, in the kidney, Panx-1 is needed for the release of intracellular ATP from renal epithelial cells [19,20]. Subsequently, there is a decrease in the ATP/adenosine ratio to control inflammation and initiation of wound-healing processes that can lead to fibrosis (see [21] for a review). Adenosine is transported through nucleoside transporters (NT), being ENT1 and CNT2 good candidates, since they show high affinity for adenosine (40 μM and 8 μM, respectively) (see [22] for a review). However, CNT2 is a Na+-dependent concentrative transporter, which makes CNT2 an ideal candidate for regulating extracellular adenosine levels. A2A receptors (A2AR) are considered the primary anti-inflammatory effectors of extracellular adenosine due to their high expression on immune cells, such as monocytes/macrophages. A2ARs mainly activate the adenylate cyclase-cAMP-PKA canonical pathway and participate in tissue remodeling and reparation. Activation of A2AR in renal macrophages induces the expression of the anti-inflammatory cytokines and reduces kidney damage in the acute and chronic inflammation phases of glomerulonephritis [23].

This study was undertaken to examine the role of purinergic pathways with respect to pretransplant inflammation of kidneys from deceased donors, and specifically to investigate the role of adenosine A2AR and how it can influence macrophage phenotype.

## 2. Results

### 2.1. Differences in Purinome Gene Expression in Renal Biopsies from DD and LD

**Figure 1:**
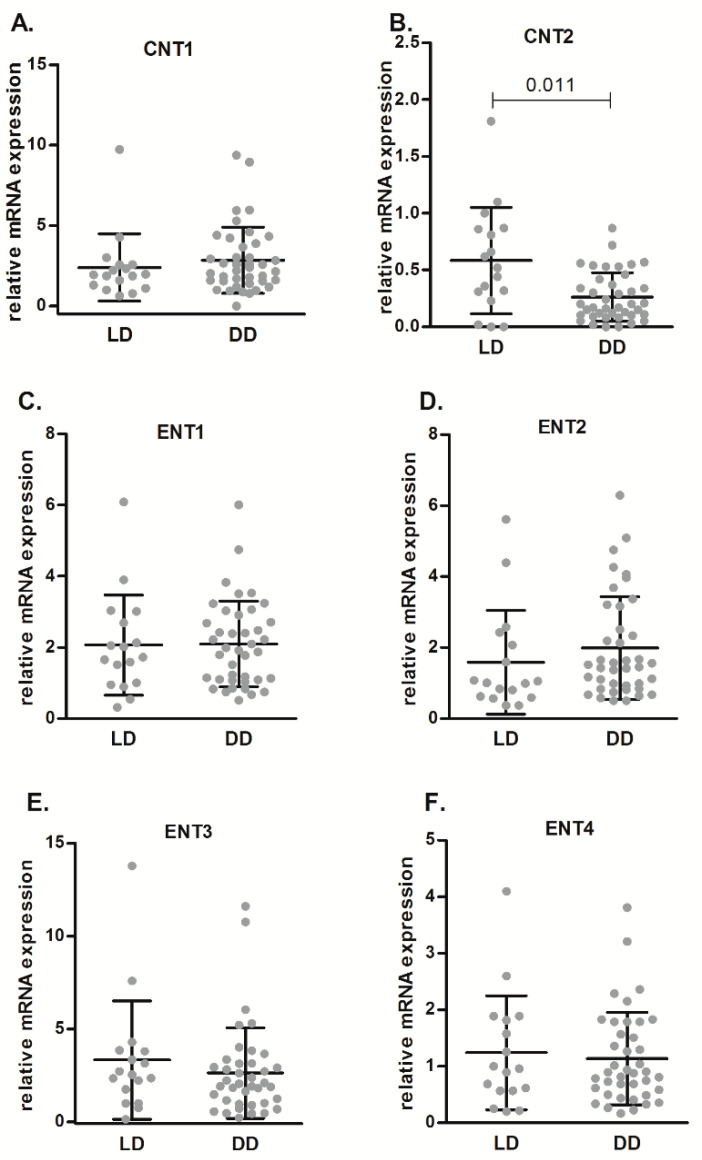
Relative mRNA expression of nucleoside transporters (CNT1 (**A**), CNT2 (**B**), ENT1 (**C**), ENT2 (**D**), ENT3 (**E**), and ENT4 (**F**)), as determined by qPCR analysis, in renal samples from LD and DD. Data are expressed as box-and-whisker plots. *p* value is shown when comparisons are statistically significant (Mann–Whitney U-test, *p* < 0.05) between groups. LD, living donors; DD, deceased donors.

Gene expression levels related to cell purinome in pre-implantation renal tissue samples were analyzed to investigate expression differences between LD and DD. Figure 1 shows that we did not find statistically significant differences between LD and DD regarding mRNA expression levels of nucleoside transporters (NTs), except for hCNT2, which shows high affinity for adenosine and showed lower basal expression in DD than in LD (*p* = 0.019). We did not detect mRNA expression of hCNT3 in renal biopsy samples (data not shown).

**Figure 2:**
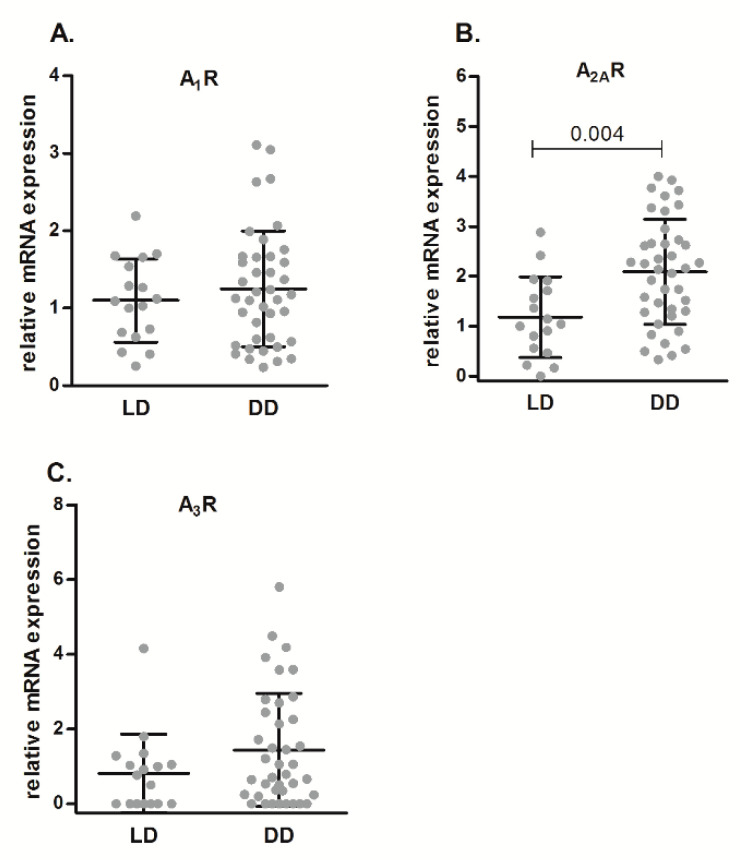
Relative mRNA expression of adenosine receptors (A1R (**A**), A2AR (**B**), and A3R (**C**)), as determined by qPCR analysis, in renal samples from LD and DD. Data are expressed as box-and-whisker plots. *p* value is shown when comparisons are statistically significant (Mann–Whitney U-test, *p* < 0.05) between groups. LD, living donors; DD, deceased donors.

Regarding the expression of P1 receptors (Figure 2), A1R (Figure 2A), and A3R (Figure 2C) did not show differences in mRNA levels between DD and LD, whereas A2AR (Figure 2B) showed higher expression levels in DD than in LD (*p* = 0.001). A2BR mRNA was hardly detected in renal biopsies samples (data not shown).

**Figure 3:**
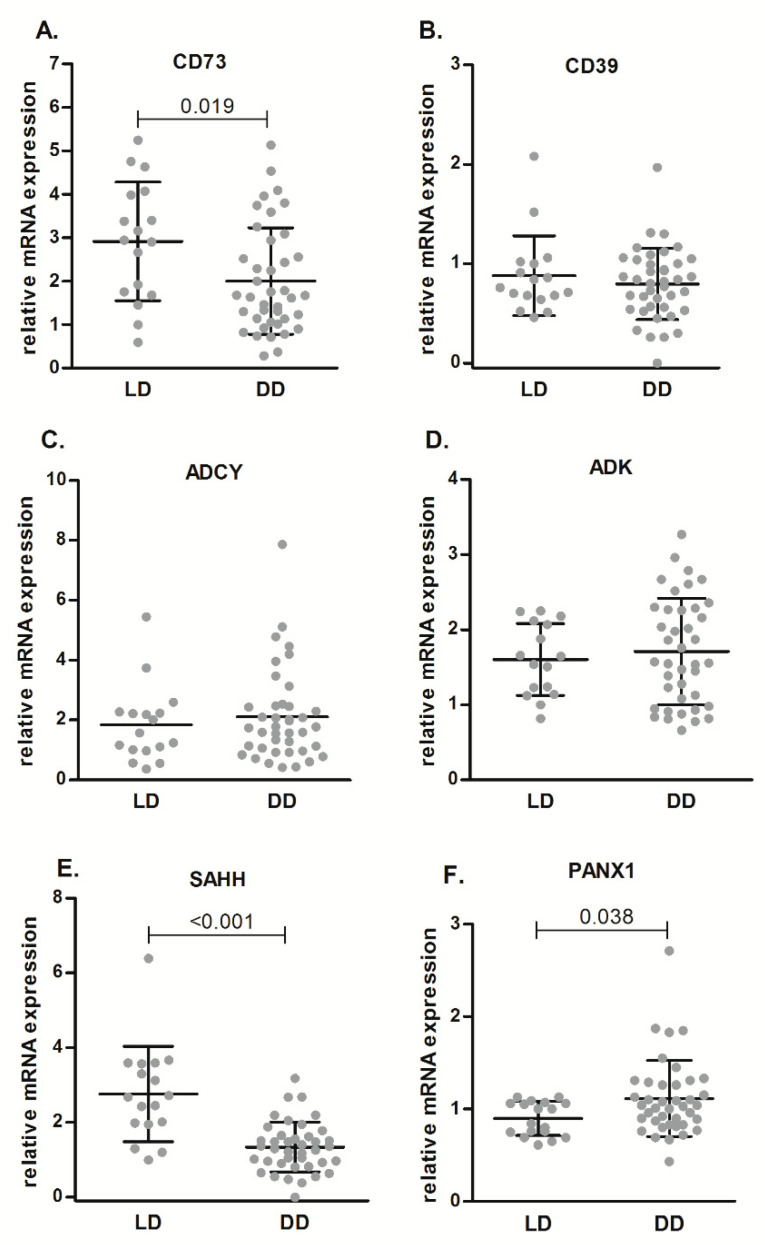
Relative mRNA expression of purinergic enzymes (CD73 (**A**), CD39 (**B**), ADCY (**C**), ADK (**D**), SAHH (**E**), PANX1 (**F**)), as determined by qPCR analysis, in renal samples from LD and DD. Data are expressed as box-and-whisker plots. *p* value is shown when comparisons are statistically significant (Mann–Whitney U-test, *p* < 0.05) between groups. LD, living donors; DD, deceased donors.

Figure 3 shows gene expression levels of different genes related to purinergic metabolism or ATP release in renal samples from LD and DD. mRNA levels of CD73 (Figure 3A) and S-adenosyl-L-homocysteine hydrolase (SAHH) (Figure 3E) was lower in DD than in LD (*p* = 0.009 and *p* < 0.001, respectively), whereas we did not observe any difference in CD39 (Figure 3B), adenylate cyclase (ADCY) (Figure 3C), or adenosine kinase (ADK) (Figure 3D) mRNA expression levels. The hemichannel Panx-1, showed higher mRNA levels in DD samples than in LD samples (*p* = 0.017) (Figure 3F).

### 2.2. Correlations of A2AR mRNA Expression Levels

To explore possible associations, correlations of gene expression levels of the different genes studied with A2AR were determined. Table 1 shows correlations (*p* and Spearman’s rho values) between A_2A_R and enzymes involved in adenosine metabolism and nucleoside transporters.

In general trends A_2A_R mRNA levels in DD samples positively correlated with the mRNA amounts of a panel of enzymes (i.e., CD73, ADK) and transporters (i.e., hENT1, hENT2, hCNT2) known to build up the cell purinome, likely to modulate adenosine-related biological events. Table 2 shows gene expression positive correlations detected between M1 macrophage phenotype or inflammatory markers (i.e., TNF-α, CD16, IL-1β), M2 macrophage phenotype or anti-inflammatory markers (i.e., IL-10, CD206, CEBPB) and fibrotic markers (i.e., TGFB1, ACTA2) with A_2A_R, in renal tissue samples of DD.

Table 2 also shows differences in expression levels of these genes between DD and LD renal samples. Expression levels of several markers related to inflammation or phenotype change of macrophages were up-regulated in DD (i.e., TNF-α, IL-1β, CD163), while of the fibrosis markers, TGF-β1 and col1A1 were the only ones overexpressed in DD. Interestingly, expression levels of A2A and TNF-α are higher in DD than in LD and correlate in both donor groups. Although no association was found between A2AR mRNA levels and factors, such as donor age or cold ischemia time, an inverse correlation was observed between the amount of PANX1 mRNA and cold ischemia time, only in kidneys from DD (*p* = 0.0023, r = −0.4522). With regard to fibrosis, we showed previously that DDs are significant older than LDs, whereas the analysis of pre-transplant kidney biopsies using the Remuzzi score, only for research purposes and not to determine the suitability of the graft, found that more than 80% of both, DD and LD samples, obtained a total score ≤ 3 [14].

### 2.3. Protein Expression in Renal Biopsies from DD and LD

**Figure 4:**
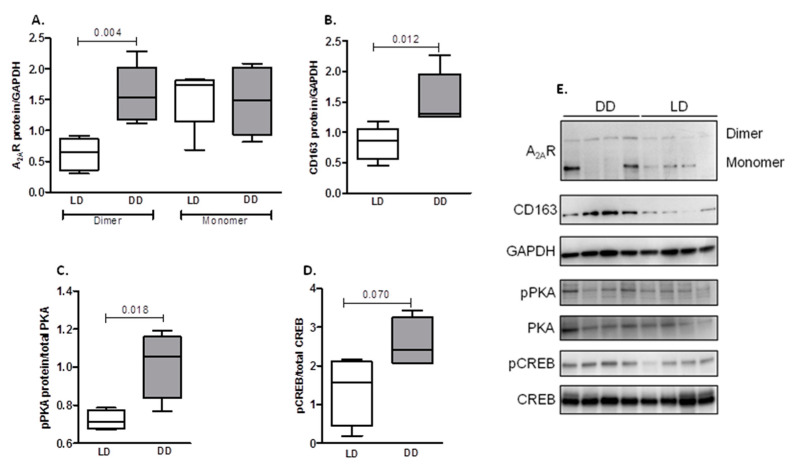
Western blot analysis of protein expression of A_2A_R (**A**), CD163 (**B**), phospho PKA (pPKA) (**C**) and phospho CREB (pCREB) (**D**) in renal samples from DD and LD. Total protein was isolated from kidney tissues, as described in Materials and Methods, and separated by SDS-PAGE. Protein expression semiquantitation performed by densitometric analysis of Western blots. (**E**) Representative Western blots for A_2A_R, CD163, pPKA and pCREB. Data are expressed as box-and-whisker plots. *p* value is shown when comparisons are statistically significant (unpaired Student’s *t*-test, *p* < 0.05) between groups. LD, living donors; DD, deceased donors.

Protein levels were measured in pre-implantation renal tissue of both DD and LD, and these data are summarized in Figure 4. It shows that the expression of the active dimer form ofA_2A_R (*p* = 0.004) (Figure 4A), CD163 (*p* = 0.012) (Figure 4B), and pPKA (*p* = 0.018) (Figure 4C) proteins was significantly higher in kidney extracts from DD compared with LD samples. Levels of pCREB were also marginally higher in DD, although statistical significance was not reached (*p* = 0.070) (Figure 4D).

### 2.4. TNF-α Increases Expression of CD163 and TGF-β1 through A2AR in THP-1 Cells

**Figure 5:**
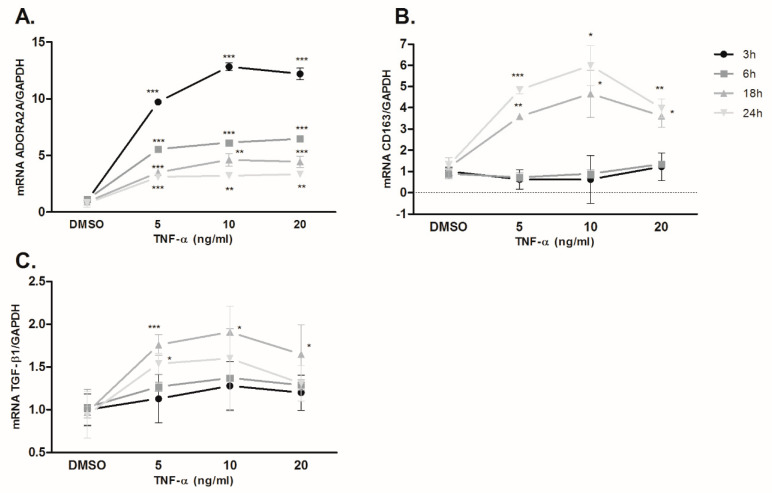
TNF-α enhances A2AR, CD163, and TGF-β1 levels in THP-1 cells. The time course of TNF-α induced A2AR (**A**), CD163 (**B**), and TGF-β1 (**C**) expression in THP-1 cells as quantitated by qPCR. *, ** and *** denote significant differences (*p* < 0.05, *p* < 0.01 and *p* < 0.001, respectively) as compared with the DMSO group using unpaired Student’s *t*-test. All values are expressed as means ± SD (*n* = 3).

THP-1 is a human leukemia monocytic cell line, which has been extensively used to study monocyte/macrophage functions. In the present study, regulation of the A_2A_R pathway and monocyte activation by TNF-α was examined using the in vitro model of undifferentiated THP-1 monocytes. TNF-α addition increased A_2A_R mRNA expression levels in THP-1 cells (Figure 5A) as previously reported [24]. This increase was significant at all time points analyzed, although the results showed a dependence on TNF-α incubation time and concentration, showing the maximum increase 3 h after 10 ng/mL TNF-α addition. CD163 and TGF-β1 gene mRNA levels were up-regulated at 18 and 24 h of treatment (Figure 5B). Shorter treatments (3 and 6 h) did not result in changes in mRNA levels.

**Figure 6:**
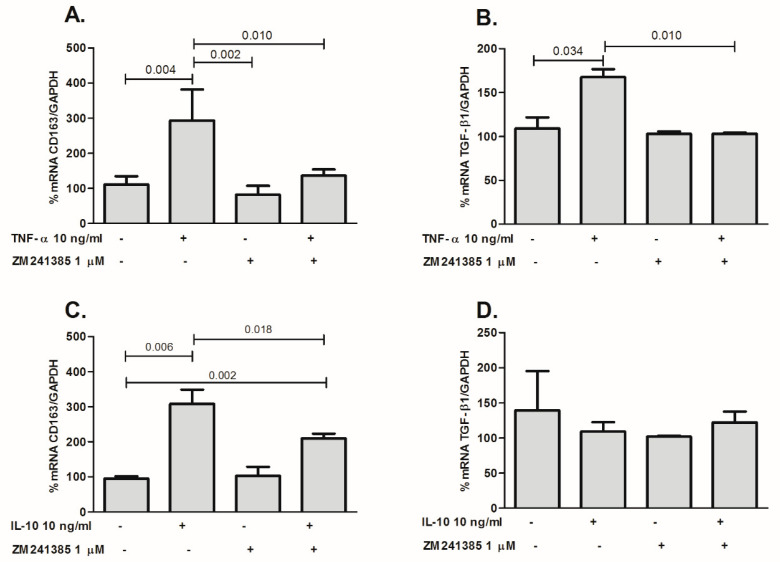
Effect of the A_2A_R receptor antagonist ZM241385 on CD163 (**A**) and TGF-β1 (**B**) gene expression induced by TNF-α in THP-1 cells. Cells were also treated with IL-10 and ZM241385 to evaluate its effect on CD163 (**C**) and TGF-β1 (**D**) gene expression. THP-1 cells were treated with ZM241385 (1 µM) 30 min before adding TNF-α (10 ng/mL) or IL-10 (10 ng/mL) for 18 h. Cells were collected, and total RNA was extracted and retrotranscribed to cDNA. Results are expressed as mean ± SD; *p* value is shown when comparisons are statistically significant (unpaired Student’s *t*-test, *p* < 0.05), *n* = 3 for each point.

Figure 6 shows that pretreatment with 1 µM ZM241385 (A_2A_R antagonist) abolished the effect of TNF-α on CD163 (Figure 6A) and TGF-β1 (Figure 6B) mRNA up-regulation, providing evidence that the A_2A_ receptor is involved in M2 macrophage activation by TNF-α. Treatment of THP-1 cells with a known CD163 inducer, IL-10, similarly increased CD163 mRNA expression levels in a manner that was also dependent upon A2AR signaling, as demonstrated by its blockade by ZM241385, showing the role of the A_2A_R in the shift to M2 phenotype (Figure 6C). There was no observed effect of IL-10 on the expression of TGF-β1 (Figure 6D).

**Figure 7:**
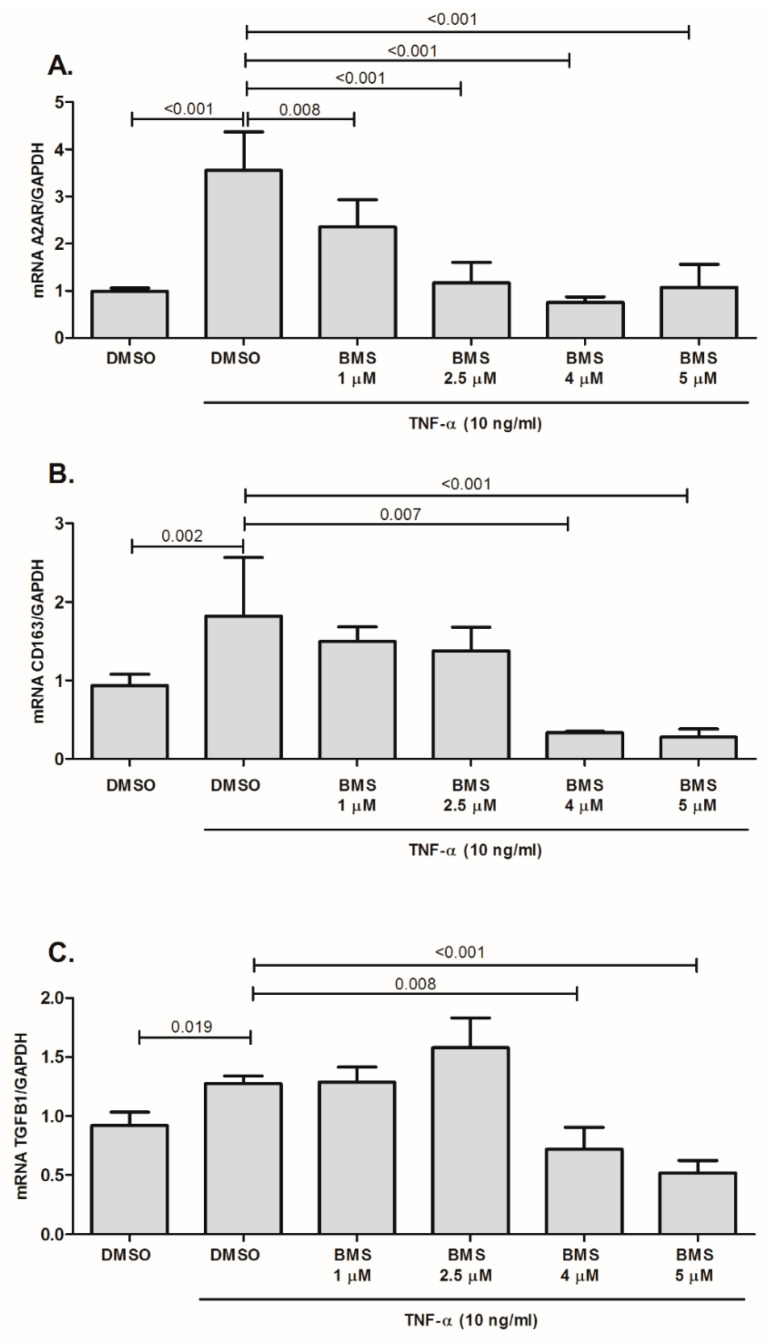
Effect of the NF-κB inhibitor BMS354451 on the A2AR (**A**), CD163 (**B**) and TGF-β1 (**C**) mRNA levels in THP-1 cells stimulated with TNF-α (10 ng/mL). BMS354451 (1, 2.5, 4 and 5 μM) was added to the cells 1 h before cytokine treatment and cultured for 20 h. Results are expressed as mean ± SD; *p* value is shown when comparisons are statistically significant (unpaired Student’s *t*-test, *p* < 0.05), *n* = 4–8 for each point.

To know the potential involvement of the NF-κB signaling-pathway in these events, cells were pre-treated with BMS345541, a highly selective inhibitor of IκB Kinase. Figure 7 shows this inhibition blocked the up-regulation of gene expression levels of A_2A_R (Figure 7A), CD163 (Figure 7B) and TGF-β1 (Figure 7C), triggered by TNF-α.

## 3. Discussion

This study highlights different activated processes in kidney allografts from DD versus LD before transplantation. Renal transplant recipients show higher concentrations of proinflammatory cytokines in grafts from DD than in those from LD [25]. We recently reported that inflammatory and reparative responses coexist in DD kidney grafts, indicating that pre-implantation kidney grafts from DD exhibit more inflammation than those from LD [14]. Inflammation can be modulated by adenosine [26,27], which exerts its anti-inflammatory activity mainly through A_2A_R [28,29,30]. Our results indicate that A_2A_R expression is increased in DD kidney grafts, which would induce anti-inflammatory responses that eventually could lead to the onset of renal fibrosis if uncontrolled or persistent inflammation occurs [31]. Monocytes and macrophages synthesize and release TNF-α, a potent mediator of inflammation that induces A_2A_R activity [24,32]. A_2A_R expression can be up-regulated directly by TNF-α [33] because of the presence of NF-κB binding sites in the upstream regions of the A_2A_R gene [24,34], but it has also been suggested that TNF-α may inhibit desensitization of A_2A_R and enhance the functions of the receptor [35].

Evidence suggests a role of TNF-α in renal interstitial fibrosis and collagen deposition [36], which may be mediated by TGF-β1 increase [37,38]. In our study, renal samples from DD showed higher gene expression of A_2A_R, TNF-α and TGF-β1 than LD and correlated positively with each other. Neutralization of TNF-α was found to reduce TGF-β1 production, myofibroblast activation, and collagen deposition, and therefore diminished renal interstitial fibrosis [39]. Our results with THP-1 cells confirm that TNF-α increases A_2A_R gene expression through the NF-κβ pathway, which can induce M2 phenotype and fibrosis.

Up-regulation of A_2A_R is likely to be the beginning of a reduction in inflammatory response. A_2A_R downregulates classic macrophage activation, reducing the production of proinflammatory cytokines such as TNF-α and increasing the expression of the anti-inflammatory cytokine IL-10 [40]. In addition, it was reported that A_2A_R antagonists promote the M1 phenotype of macrophages infiltrating nephritic glomeruli [41]. A_2A_R signaling through G proteins is based on the stimulation of adenylate cyclase (AC), which induces intracellular cAMP and activates protein kinase A (PKA). Moreover, A_2A_R and AC mRNA expression showed a positive correlation in DD samples and p-PKA was increased in DD samples compared with LD samples. Chronic inflammatory tissue injury is accompanied by the accumulation of extracellular adenosine released by immune and non-immune cells. Adenosine receptor expression in macrophages appears to change upon inflammatory activation, since A_2A_Rfunction in human THP-1 monocytes has been shown to be up-regulated by IL-1β and TNF-α [24]. Indeed, our results show that A_2A_R gene expression in THP-1 monocytes is rapidly up-regulated by TNF-α, suggesting that anti-inflammatory A_2A_R is up-regulated following classic activation of macrophages, possibly to initiate resolution of inflammation [26].

It is worth noting that the induction of A_2A_R by TNF-α is relatively fast and persistent whereas induction of CD163 and TGF-β1 occurs later and is A_2A_R-dependent. These results support that fibrotic processes are initiated through A_2A_R-activation when monocytes/macrophages are exposed to prolonged inflammation. Although CD73 expression is lower in DD than in LD samples, indicating that extracellular synthesis of adenosine might be somehow reduced, hCNT2 expression is downregulated in DD samples, which is consistent with reduced adenosine clearance from the extracellular milieu. Indeed, as recently reviewed [22], hCNT2 may be a major player in regulating extracellular adenosine levels and there is evidence of CNT2 being regulated at the post-translational level [42]. Interestingly, the hCNT2-encoding gene (SLC28A2) is by far the most dramatically down-regulated one among the purinome-related genes in inflamed ileal mucosa samples from Crohn’s disease patients [43]. Furthermore, A_2A_Rs are known to up-regulate CNT2 activity in differentiated PC12 cells [43]. Moreover, it was also observed that hypoxia downregulated CNT2 function without affecting ENT1 activity, which suggests, as mentioned above, an important role of CNT2 in the modulation of extracellular adenosine concentrations [43,44]. In this regard, A_2A_R signaling again emerges as an important mechanism for limiting inflammatory responses [45,46,47]. Although some authors observed that CD73 expression and function were upregulated by proinflammatory mediators [48,49], Zanin et al. provided evidence that proinflammatory M1 macrophages decrease both the expression and the activity of CD39 and CD73, leading to reduced ATP degradation [50]. Moreover, TNF-α was found to reduce the surface expression and activity of CD73 [51]. By contrast, M2 macrophages showed increased expression and activity of both enzymes, followed quickly by the conversion of ATP into adenosine. Our findings on pre-implant kidney grafts from DD confirmed overexpression of TNF-α and decreased expression of CD73. Chronic lack of CD73 was associated with an autoimmune inflammatory phenotype, which at the renal level affects the glomerular endothelium, leading to glomerular inflammation, injury, and interstitial cellular infiltrate, with consequent proteinuria and decreased kidney function [52]. Our results also show a little—but significantly increased—expression of Panx-1 and an inverse correlation with ischemic time in kidneys from DD. This could be due to a known regulation of the channel by the released ATP itself that can bind to a binding site in aPanx-1 extracellular loop to prevent persistent excitatory signaling of Panx1-mediated ATP release [53]. Moreover, increased extracellular ATP levels can induce the internalization of Panx1, avoiding further release of excitatory ATP [54].

In our study, low intracellular SAHH gene expression in DD grafts may contribute to the accumulation of *S*-adenosylhomocysteine (SAH) and to a decrease in intracellular adenosine and homocysteine production. Barroso et al. showed that an excess of SAH in response to NF-kB activation leads to the expression of adhesion molecules and cytokines such as IL-1β and TNF-α in endothelial cells, resulting in an inflammatory response [55].

During an inflammatory process, adenosine can activate A_2A_R to attenuate inflammation and tissue injury. Adenosine reduces the M1 macrophage phenotype and activation of A_2A_R shifts macrophages towards the M2 phenotype [16,56], and promotes wound healing in mice [57,58]. Thus, extracellular adenosine appears to facilitate a macrophage switch characteristic for an alternatively activated phenotype [59]. We have shown that A_2A_RmRNA expression correlates with the M2 macrophage marker CD163 and that both proteins are augmented in DD samples compared with LD. Our results are in accordance with the hypothesis that the onset of anti-inflammatory activity induces the M2 phenotype and is driven by A_2A_R signaling.

Stimulation of IL-10 production in RAW264.7 macrophages by adenosine was regulated by the transcription factor C/EBPβ [60]. In our study, A_2A_R expression in DD correlated with C/EBPβ and IL-10. During macrophage activation, C/EBPβ is induced by the CREB transcriptional activator, and Ruffell and co-workers found that deletion of CREB-binding sites from *Cebpb* promoter avoided macrophage activation and blocked specific M2 genes [61]. Our results suggest the activation of CREB, since phospho-CREB is up-regulated in DD renal biopsies, although statistical significance was not reached. Other models support involvement of C/EBPB in M2 macrophage differentiation and fibrosis after damage [62,63,64].

**Figure 8:**
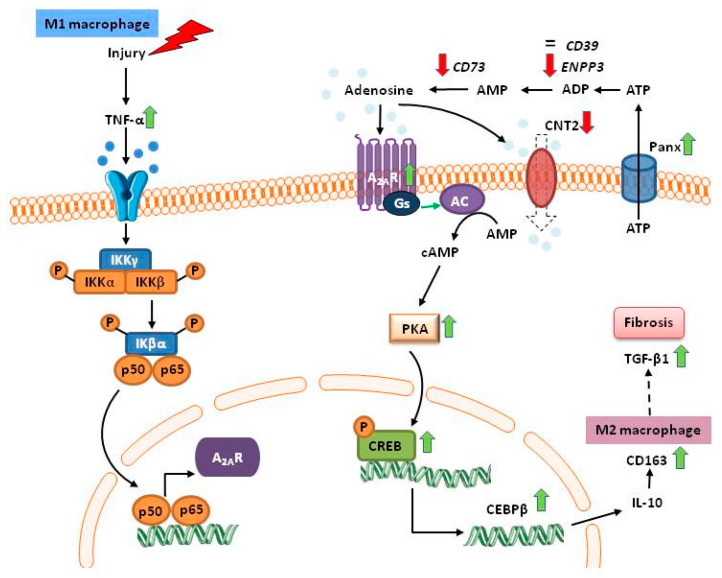
Schematic representation of the proposed mechanism of inflammation/anti-inflammation in DD kidneys. After an injury, such as hypoxia or ischemia, ATP is released and the production of the inflammatory cytokine TNF-α is induced. Via the NF-κB pathway, TNF-α stimulates A_2A_R expression although the formation and uptake of extracellular adenosine are restricted. Thus, the cAMP-PKA-CREB signaling pathway could be initiated and promote anti-inflammatory immune responses, e.g., through the induction of IL-10 and the switch to the M2 macrophage phenotype (CD163). If injury or inflammation persists, this can lead to the generation of TGF-β1, which may mediate the onset of fibrosis.

In summary, DD kidney grafts display more inflammation than LD and produce higher levels of TNF-α. Our results suggest that CD163 and TGF-β1 are regulated, at least in part, by TNF-α via A_2A_R to initiate anti-inflammatory processes and promote M2 macrophage phenotype. Our work also indicates that, in kidney biopsies from DD, the cAMP/PKA/CREB/C/EBPβ pathway could be activated, which would also favor the switch from macrophages M1 to M2. A proposed integrated mechanistic model explaining the dual inflammatory and anti-inflammatory conditions of DD kidneys is shown in Figure 8.

Although further research is needed, our results unequivocally show differences in purinergic signaling in grafts from DD and from LD which highlights the possibility of targeting purinome elements for therapeutic management of early and persistent inflammation associated with renal graft dysfunction. Adenosine signaling is considered protective in ischemic lesions with immunomodulatory properties. Our observations, although focused on kidney transplantation, show crucial changes in the immunomodulation of transplanted organs, highlighting a key role for the purinome in kidney graft inflammation and the onset of fibrosis. Our results confirm the growing evidence that purinergic signaling is involved in the inflammatory response that can be associated with rejection and chronic allograft dysfunction. Our contribution also raises the possibility of purinergic biology playing a more general role in the clinics derived from organ transplantation in humans.

## 4. Materials and Methods

### 4.1. Donors, Patients, and Kidney Samples

This study was approved by the ethics committee of Fundació Puigvert. Participants were from the study population for a previous work [14] in which patients characteristics are described [14]. In brief, 94 renal donors (60DD/34LD) were included. We obtained renal biopsies for gene expression analysis from 47 DD and 21 LD. Two living donors’ samples were missing. We collected pre-implantation biopsies from kidney donors and clinical information for donors and KTR who underwent transplantation in our institution between 2008 and 2011. All of them signed an informed consent form. Tissue samples from biopsy cores were processed for mRNA extraction and gene expression analysis by qPCR. Total protein was also obtained for Western blot.

### 4.2. Cell Culture and Treatments

Human monocytic leukemia THP-1 cells (ATCC) were cultured in RPMI (Lonza, Bassel, Switzerland) supplemented with 10% fetal bovine serum (Life Technologies, Carlsbad, CA, USA), 2 mM glutamine, and antibiotic (Lonza). Cells were grown in complete medium and then growth arrested in serum-free medium for 24 h. All experiments were performed under serum-free conditions. THP-1 cells were seeded at 37 °C in a humidified 5% CO_2_/95% air atmosphere in the presence of 5, 10, and 20 ng/mL of TNF-α (R&D Systems, Minneapolis, MN, USA) at different time points (3, 6, 18, and 24 h). When needed and at the times selected, cells were pretreated for 30 min with 1µM of the A_2A_R antagonist ZM241385 (Tocris, Bristol, UK) or for 60 min with the NF-κβ blocker BMS345541 (Sigma, Saint Louis, MO, USA) (1, 2.5, 4, and 5 μM) before the addition of TNF-α. Control samples always contained the same amount of vehicle (DMSO) to exclude any interference in cell responses. After the incubation period, cells were centrifuged and pellets were resuspended in TriReagent (Sigma).

### 4.3. Real Time PCR

#### 4.3.1. Renal Tissue

Kidney biopsies were processed as described previously [14]. Briefly, samples were homogenized with Tissuelyser LT (Qiagen, Hilden, Germany). Aqueous phase containing RNA was transferred into an RNeasy column (AllPrep DNA/RNA/Protein Mini Kit, Qiagen) and was eluted with RNAse-free water. The integrity of total RNA was assessed on a denaturing agarose gel, allowing visual assessment of the 28S and 18S rRNA bands. Retrotranscription of total RNA to cDNA was done according to the user guide of the Applied Biosystems™ QuantStudio™ 12K Flex Real-Time PCR System, OpenArray^®^ Experiments (Life Technologies, Carlsbad, CA, USA). Briefly, 10 µL of 2X reverse transcription mix of the High Capacity cDNA Reverse Transcription kit (Life Technologies) was mixed with 10 µL of total RNA in a 96-well reaction plate. Plates were incubated at room temperature for 10 min, then incubated at 37 °C for 2 h and placed on ice for 5 min, incubated at 75 °C for 10 min, placed on ice for 5 min, and spun down. Real-time PCR was done in accordance with the TaqMan^®^gene expression assays protocol (Life Technologies). A mix containing 2X TaqMan OpenArray Real Time PCR Master Mix (Cat No. 4.462.159 Life Technologies) and cDNA was loaded on each OpenArray plate. These plates were run on the computer QuantStudio 12 K Flex Real-Time PCR system. Relative quantification of gene expression was performed using the expression of three internal controls: human GAPDH, β-actin, and β-glucuronidase.

#### 4.3.2. THP-1 Cells

Total RNA was isolated from THP-1 cells with TriReagent (Sigma, St. Louis, MO, USA) and 1 µg RNA was retrotranscribed to cDNA with the MultiScribe Reverse Transcriptase kit (Applied Biosystems, Carlsbad, CA, USA) according to the manufacturer’s instructions. Real-time amplification of cDNAs was carried out with the TaqMan Universal Master Mix (Applied Biosystems) in the StepOne Sequence Detection System (Applied Biosystems). Assays used for amplification of human CD163, A2AR, and TGF-β1 were predesigned inventoried TaqMan Gene Expression Assays (Applied Biosystems, Foster City, CA, USA). Relative quantification of gene expression was performed as described in the TaqMan instruction manual using human GAPDH as an internal control. The PCR arbitrary units of the genes analyzed were defined as the mRNA levels of these genes normalized to the GAPDH expression level in order to quantify these transcripts in relative terms.

### 4.4. Western Blot

Protein samples were obtained from kidney biopsies with AllPrep DNA/RNA/Protein Mini Kit (Qiagen, Venlo, The Netherlands). Total protein pellets were dissolved in 8M urea lysis buffer, centrifuged to eliminate cell debris, and supernatant was stored to −80 °C.

Thirty micrograms of total proteins were fractionated by SDS-PAGE and transferred to a polyvinylidene difluoride membrane using a Trans-blot turbo (Bio-Rad, Hercules, CA, USA). After incubation with 5% BSA in TBST (10 mM Tris, pH 8.0, 150 mM NaCl, 0.5% Tween 20), the membrane was washed with TBST and incubated with antibodies against phospho (p)-CREB (Cell Signaling, Danvers, MA, USA; dilution 1/1000), CREB (Cell Signaling; 1/500), phospho (p)-PKA (Cell Signaling; 1/1000), PKA (Cell Signaling; 1/500), A_2A_R(St John’s Laboratory, London, UK; 1/500), CD163 (Pierce, Waltham, MA, USA; 1/1000), and GAPDH (Sta. Cruz Biotechnology, Santa Cruz, CA, USA; 1/2000), the last as loading control, at 4 °C O/N under agitation. Membranes were washed with TBST and incubated with a 1:20,000 dilution of horseradish peroxidase-conjugated anti-mouse or anti-rabbit antibody (Bio-Rad, Hercules, CA, USA) for 1 h. Membranes were washed again and blots were exposed to highly sensitive films and developed using an ECL^®^ technique (Pierce).

### 4.5. Statistics

GraphPad Prism software (GraphPad Software, San Diego, CA, USA)was used to perform statistical analysis. Results are expressed as the mean ± standard deviation (lower and upper extremities) and percentages, as appropriate. Student’s *t*-test was applied to compare means. When our data did not follow a Gaussian distribution, nonparametric tests such as the Mann–Whitney U test and Spearman correlation were used for data analysis. All *p* values were two-sided, and *p* < 0.05 was considered significant. Spearman test significance was confirmed by *p*-value analysis using the Benjamini–Hochberg procedure with a criterion of <5% False Discovery Rate [65].

## Figures and Tables

**Table 1:** Gene expression correlations between A_2A_R and enzymes involved in adenosine metabolism and nucleoside transporters.

		A2AR Correlations			
		DD		LD	
		*p*	Rho Spearman	*p*	Rho Spearman
Enzymes	CD39	0.065	0.272	0.311	0.246
	CD73	<0.001	0.635	0.705	−0.093
	ADK	<0.001	0.609	0.433	0.191
	SAHH	<0.001	0.501	0.697	0.096
	ADCY	<0.001	0.521	0.271	0.266
Nucleoside transporters	ENT1	<0.001	0.603	0.170	0.328
	ENT2	0.003	0.426	0.507	0.162
	ENT3	<0.001	0.563	0.673	0.104
	ENT4	0.002	0.441	0.459	0.181
	CNT1	0.049	0.288	0.792	0.065
	CNT2	0.017	0.347	0.665	−0.106

LD, living donors; DD, deceased donors.

**Table 2:** RNA expression levels of different genes in DD vs. LD and gene expression Spearman rank correlations coefficients between A_2A_R and inflammatory, anti-inflammatory and fibrosis genes.

				A2AR Correlations			
		RNA Expression		DD		LD	
		DD vs. LD	Significance	*p*	Rho Spearman	*p*	Rho Spearman
Inflammatory & M1 markers	TNF	↑	[14] *	<0.001	0.504	0.022	0.520
	NFKB	↑	*p* = 0.033	<0.001	0.626	0.997	0.001
	CD16	↑	[14] *	<0.001	0.507	0.131	0.359
	CD86	=	[14] *	<0.001	0.478	0.303	0.250
	IL-1β	↑	[14] *	0.003	0.432	0.983	0.005
Anti-inflammatory & M2 markers	IL10	=	n.s.	<0.001	0.492	0.265	0.269
	CD206	=	[14] *	<0.001	0.577	0.132	0.358
	CD163	↑	[14] *	<0.001	0.502	0.209	0.302
	IL13RA2	=	n.s.	0.002	0.437	0.204	0.305
	CD209	↑	[14] *	0.056	0.281	0.557	0.144
	CEBPB	↑	*p* < 0.001	<0.001	0.482	0.446	0.186
EMT & Fibrosis markers	TGFB1	↑	[14] *	<0.001	0.712	0.061	0.437
	FIBRONECTIN	=	[14] *	<0.001	0.652	0.545	0.148
	ACTA2	=	[14] *	<0.001	0.572	0.123	0.366
	VIMENTIN	=	[14] *	0.002	0.447	0.718	0.089
	COL1A1	↑	*p* = 0.006	0.005	0.407	0.102	0.386
	COL1A2	=	n.s.	<0.001	0.511	0.158	0.337
	COL3A1	=	n.s.	0.015	0.354	0.051	0.454

Significance is shown as the value of “*p*” when lower than 0.05 or as n.s. (not significant) when higher according to the Mann–Whitney U test. [14] * corresponds to the reference of the previous study in which the gene was included and analyzed. LD, living donors; DD, deceased donors.

## Abbreviations

DD	Deceased donor
LD	Living donor
A_2A_R	A2A adenosine receptor
KTR	Kidney transplant recipient
NT	Nucleoside transporter
SAHH	S-adenosyl-L-homocysteine hydrolase
AC	Adenylate cyclase
ADK	Adenosine kinase
PKA	Protein kinase A
SAH	S-adenosylhomocysteine
ENT	Equilibrative nucleoside transporter
CNT	Concentrative nucleoside transporter
hENT	Human ENT
hCNT	Human CNT

## References

[1] Opelz G. (1997). Impact of HLA compatibility on survival of kidney transplants from unrelated live donors. Transplantation.

[2] Moreso F., Serón D., Gil-Vernet S., Riera L., Fulladosa X., Ramos R., Alsina J., Grinyó J.M. (1999). Donor age and delayed graft function as predictors of renal allograft survival in rejection-free patients. Nephrol. Dial. Transpl..

[3] Snoeijs M.G.J., van Bijnen A., Swennen E., Haenen G.R.M.M., Roberts L.J., Christiaans M.H.L., Peppelenbosch A.G., Buurman W.A., Ernest van Heurn L.W. (2011). Tubular epithelial injury and inflammation after ischemia and reperfusion in human kidney transplantation. Ann. Surg..

[4] Pessione F., Cohen S., Durand D., Hourmant M., Kessler M., Legendre C., Mourad G., Noël C., Peraldi M.-N., Pouteil-Noble C. (2003). Multivariate analysis of donor risk factors for graft survival in kidney transplantation. Transplantation.

[5] van der Hoeven J.A., Ploeg R.J., Postema F., Molema I., de Vos P., Girbes A.R., van Suylichem P.T., van Schilfgaarde R., Ter Horst G.J. (1999). Induction of organ dysfunction and up-regulation of inflammatory markers in the liver and kidneys of hypotensive brain dead rats: A model to study marginal organ donors. Transplantation.

[6] van der Hoeven J.A.B., Molema G., Ter Horst G.J., Freund R.L., Wiersema J., van Schilfgaarde R., Leuvenink H.G.D., Ploeg R.J. (2003). Relationship between duration of brain death and hemodynamic (in)stability on progressive dysfunction and increased immunologic activation of donor kidneys. Kidney Int..

[7] Nijboer W.N., Schuurs T.A., van der Hoeven J.A.B., Fekken S., Wiersema-Buist J., Leuvenink H.G.D., Hofker S., Homan van der Heide J.J., van Son W.J., Ploeg R.J. (2004). Effect of brain death on gene expression and tissue activation in human donor kidneys. Transplantation.

[8] Nagareda T., Kinoshita Y., Tanaka A., Takeda M., Sakano T., Yawata K., Sugimoto T., Nishizawa Y., Terada N. (1993). Clinicopathology of kidneys from brain-dead patients treated with vasopressin and epinephrine. Kidney Int..

[9] Kreisel D., Krupnick A.S., Gelman A.E., Engels F.H., Popma S.H., Krasinskas A.M., Balsara K.R., Szeto W.Y., Turka L.A., Rosengard B.R. (2002). Non-hematopoietic allograft cells directly activate CD8+ T cells and trigger acute rejection: An alternative mechanism of allorecognition. Nat. Med..

[10] Pratschke J., Wilhelm M.J., Laskowski I., Kusaka M., Beato F., Tullius S.G., Neuhaus P., Hancock W.W., Tilney N.L. (2001). Influence of donor brain death on chronic rejection of renal transplants in rats. J. Am. Soc. Nephrol..

[11] Guillén-Gómez E., Guirado L., Belmonte X., Maderuelo A., Santín S., Juarez C., Ars E., Facundo C., Ballarín J.A., Vidal S. (2014). Monocyte implication in renal allograft dysfunction. Clin. Exp. Immunol..

[12] Magil A.B. (2009). Monocytes/macrophages in renal allograft rejection. Transpl. Rev. (Orlando).

[13] Serón D., O’Valle F., Moreso F., Gomà M., Hueso M., Grinyó J.M., Garcia del Moral R. (2007). Immunophenotype of infiltrating cells in protocol renal allograft biopsies from tacrolimus-versus cyclosporine-treated patients. Transplantation.

[14] Guillén-Gómez E., Dasilva I., Silva I., Arce Y., Facundo C., Ars E., Breda A., Ortiz A., Guirado L., Ballarín J.A. (2017). Early Macrophage Infiltration and Sustained Inflammation in Kidneys From Deceased Donors Are Associated With Long-Term Renal Function. Am. J. Transpl..

[15] Csoka B., Selmeczy Z., Koscso B., Nemeth Z.H., Pacher P., Murray P.J., Kepka-Lenhart D., Morris S.M., Gause W.C., Leibovich S.J. (2012). Adenosine promotes alternative macrophage activation via A2A and A2B receptors. FASEB J..

[16] Pinhal-Enfield G., Ramanathan M., Hasko G., Vogel S.N., Salzman A.L., Boons G.-J., Leibovich S.J. (2003). An Angiogenic Switch in Macrophages Involving Synergy between Toll-Like Receptors 2, 4, 7, and 9 and Adenosine A2A Receptors. Am. J. Pathol..

[17] Kurashima Y., Amiya T., Nochi T., Fujisawa K., Haraguchi T., Iba H., Tsutsui H., Sato S., Nakajima S., Iijima H. (2012). Extracellular ATP mediates mast cell-dependent intestinal inflammation through P2 × 7 purinoceptors. Nat. Commun..

[18] Crespo Yanguas S., Willebrords J., Johnstone S.R., Maes M., Decrock E., De Bock M., Leybaert L., Cogliati B., Vinken M. (2017). Pannexin1 as mediator of inflammation and cell death. Biochim. Biophys. Acta Mol. Cell Res..

[19] Hanner F., Lam L., Nguyen M.T.X., Yu A., Peti-Peterdi J. (2012). Intrarenal localization of the plasma membrane ATP channel pannexin1. Am. J. Physiol. Ren. Physiol..

[20] Jankowski J., Perry H.M., Medina C.B., Huang L., Yao J., Bajwa A., Lorenz U.M., Rosin D.L., Ravichandran K.S., Isakson B.E. (2018). Epithelial and Endothelial Pannexin1 Channels Mediate AKI. J. Am. Soc. Nephrol..

[21] Cekic C., Linden J. (2016). Purinergic regulation of the immune system. Nat. Rev. Immunol..

[22] Pastor-Anglada M., Pérez-Torras S. (2018). Who is who in Adenosine transport. Front. Pharmacol..

[23] Garcia G.E., Truong L.D., Li P., Zhang P., Du J., Chen J.-F., Feng L. (2008). Adenosine A2A receptor activation and macrophage-mediated experimental glomerulonephritis. FASEB J..

[24] Khoa N.D., Montesinos M.C., Reiss A.B., Delano D., Awadallah N., Cronstein B.N. (2001). Inflammatory cytokines regulate function and expression of adenosine A(2A) receptors in human monocytic THP-1 cells. J. Immunol..

[25] Koo D.D.H., Welsh K.I., McLaren A.J., Roake J.A., Morris P.J., Fuggle S.V. (1999). Cadaver versus living donor kidneys: Impact of donor factors on antigen induction before transplantation. Kidney Int..

[26] Bours M.J.L., Swennen E.L.R., Di Virgilio F., Cronstein B.N., Dagnelie P.C. (2006). Adenosine 5???-triphosphate and adenosine as endogenous signaling molecules in immunity and inflammation. Pharm. Ther..

[27] Lawrence T., Willoughby D.A., Gilroy D.W. (2002). Anti-inflammatory lipid mediators and insights into the resolution of inflammation. Nat. Rev. Immunol..

[28] Haskó G., Pacher P., Deitch E.A., Vizi E.S. (2007). Shaping of monocyte and macrophage function by adenosine receptors. Pharm. Ther..

[29] Haskó G., Kuhel D.G., Chen J.F., Schwarzschild M.A., Deitch E.A., Mabley J.G., Marton A., Szabó C. (2000). Adenosine inhibits IL-12 and TNF-[alpha] production via adenosine A2a receptor-dependent and independent mechanisms. FASEB J..

[30] Buenestado A., Grassin Delyle S., Arnould I., Besnard F., Naline E., Blouquit-Laye S., Chapelier A., Bellamy J.F., Devillier P. (2010). The role of adenosine receptors in regulating production of tumour necrosis factor-alpha and chemokines by human lung macrophages. Br. J. Pharm..

[31] Lee S.B., Kalluri R. (2010). Mechanistic connection between inflammation and fibrosis. Kidney Int. Suppl..

[32] Morello S., Ito K., Yamamura S., Lee K.-Y., Jazrawi E., DeSouza P., Barnes P., Cicala C., Adcock I.M. (2006). IL-1 and TNF- Regulation of the Adenosine Receptor (A2A) Expression: Differential Requirement for NF- B Binding to the Proximal Promoter. J. Immunol..

[33] Capecchi P.L., Camurri A., Pompella G., Mazzola A., Maccherini M., Diciolla F., Lazzerini P.E., Abbracchio M.P., Laghi-Pasini F. (2005). Upregulation of A2A adenosine receptor expression by TNF-alpha in PBMC of patients with CHF: A regulatory mechanism of inflammation. J. Card. Fail..

[34] St Hilaire C., Carroll S.H., Chen H., Ravid K. (2009). Mechanisms of induction of adenosine receptor genes and its functional significance. J. Cell. Physiol..

[35] Khoa N.D., Postow M., Danielsson J., Cronstein B.N. (2006). Tumor necrosis factor-alpha prevents desensitization of Galphas-coupled receptors by regulating GRK2 association with the plasma membrane. Mol. Pharm..

[36] Bani-Hani A.H., Campbell M.T., Meldrum D.R., Meldrum K.K. (2008). Cytokines in epithelial-mesenchymal transition: A new insight into obstructive nephropathy. J. Urol..

[37] Bates R.C., Mercurio A.M. (2003). Tumor necrosis factor-alpha stimulates the epithelial-to-mesenchymal transition of human colonic organoids. Mol. Biol. Cell.

[38] Meldrum K.K., Misseri R., Metcalfe P., Dinarello C.A., Hile K.L., Meldrum D.R. (2007). TNF-alpha neutralization ameliorates obstruction-induced renal fibrosis and dysfunction. Am. J. Physiol. Regul. Integr. Comp. Physiol..

[39] Therrien F.J.F.J., Agharazii M., Lebel M., Larivière R., Larivire R. (2012). Neutralization of tumor necrosis factor-alpha reduces renal fibrosis and hypertension in rats with renal failure. Am. J. Nephrol..

[40] Haskó G., Pacher P. (2012). Regulation of macrophage function by adenosine. Arter. Thromb. Vasc. Biol..

[41] Garcia G.E., Truong L.D., Chen J.-F., Johnson R.J., Feng L. (2011). Adenosine A(2A) receptor activation prevents progressive kidney fibrosis in a model of immune-associated chronic inflammation. Kidney Int..

[42] Fernández-Veledo S., Huber-Ruano I., Aymerich I., Duflot S., Casado F.J., Pastor-Anglada M. (2006). Bile acids alter the subcellular localization of CNT2 (concentrative nucleoside cotransporter) and increase CNT2-related transport activity in liver parenchymal cells. Biochem. J..

[43] Pérez-Torras S., Iglesias I., Llopis M., Lozano J.J., Antolín M., Guarner F., Pastor-Anglada M. (2016). Transportome Profiling Identifies Profound Alterations in Crohn’s Disease Partially Restored by Commensal Bacteria. J. Crohn’s Colitis.

[44] Medina-Pulido L., Molina-Arcas M., Justicia C., Soriano E., Burgaya F., Planas A.M., Pastor-Anglada M. (2013). Hypoxia and P1 receptor activation regulate the high-affinity concentrative adenosine transporter CNT2 in differentiated neuronal PC12 cells. Biochem. J..

[45] Ohta A., Sitkovsky M. (2001). Role of G-protein-coupled adenosine receptors in downregulation of inflammation and protection from tissue damage. Nature.

[46] Sitkovsky M.V., Lukashev D., Apasov S., Kojima H., Koshiba M., Caldwell C., Ohta A., Thiel M. (2004). Physiological control of immune response and inflammatory tissue damage by hypoxia-inducible factors and adenosine A2A receptors. Annu. Rev. Immunol..

[47] Thiel M., Chouker A., Ohta A., Jackson E., Caldwell C., Smith P., Lukashev D., Bittmann I., Sitkovsky M. (2005). V Oxygenation inhibits the physiological tissue-protecting mechanism and thereby exacerbates acute inflammatory lung injury. PLoS Biol..

[48] Beavis P.A., Stagg J., Darcy P.K., Smyth M.J. (2012). CD73: A potent suppressor of antitumor immune responses. Trends Immunol..

[49] Regateiro F.S., Howie D., Nolan K.F., Agorogiannis E.I., Greaves D.R., Cobbold S.P., Waldmann H. (2011). Generation of anti-inflammatory adenosine by leukocytes is regulated by TGF-β. Eur. J. Immunol..

[50] Zanin R.F., Braganhol E., Bergamin L.S.L.S., Campesato L.F.I.L.F.I., Filho A.Z., Moreira J.C.F.J.C.F., Morrone F.B., Sévigny J., Schetinger M.R.C., de Souza Wyse A.T. (2012). Differential macrophage activation alters the expression profile of NTPDase and Ecto-5’-nucleotidase. PLoS ONE.

[51] Kalsi K., Lawson C., Dominguez M., Taylor P., Yacoub M.H., Smolenski R.T. (2002). Regulation of ecto-5′-nucleotidase by TNF-alpha in human endothelial cells. Mol. Cell. Biochem..

[52] Blume C., Felix A., Shushakova N., Gueler F., Falk C.S., Haller H., Schrader J. (2012). Autoimmunity in CD73/Ecto-5′-nucleotidase deficient mice induces renal injury. PLoS ONE.

[53] Qiu F., Dahl G. (2009). A permeant regulating its permeation pore: Inhibition of pannexin 1 channels by ATP. Am. J. Physiol. Cell Physiol..

[54] Whyte-Fagundes P., Zoidl G. (2018). Mechanisms of pannexin1 channel gating and regulation. Biochim. Biophys. Acta Biomembr..

[55] Barroso M., Kao D., Blom H.J., Tavares de Almeida I., Castro R., Loscalzo J., Handy D.E. (2016). S-adenosylhomocysteine induces inflammation through NFkB: A possible role for EZH2 in endothelial cell activation. Biochim. Biophys. Acta Mol. Basis Dis..

[56] Ferrante C.J., Pinhal-Enfield G., Elson G., Cronstein B.N., Hasko G., Outram S., Leibovich S.J. (2013). The Adenosine-Dependent Angiogenic Switch of Macrophages to an M2-Like Phenotype is Independent of Interleukin-4 Receptor Alpha (IL-4Rα) Signaling. Inflammation.

[57] Montesinos M.C., Gadangi P., Longaker M., Sung J., Levine J., Nilsen D., Reibman J., Li M., Jiang C.K., Hirschhorn R. (1997). Wound healing is accelerated by agonists of adenosine A2 (G alpha s-linked) receptors. J. Exp. Med..

[58] Leibovich S.J., Chen J.-F., Pinhal-Enfield G., Belem P.C., Elson G., Rosania A., Ramanathan M., Montesinos C., Jacobson M., Schwarzschild M.A. (2002). Synergistic up-regulation of vascular endothelial growth factor expression in murine macrophages by adenosine A(2A) receptor agonists and endotoxin. Am. J. Pathol..

[59] Olah M.E., Caldwell C.C. (2003). Adenosine receptors and mammalian toll-like receptors: Synergism in macrophages. Mol. Interv..

[60] Csóka B., Németh Z.H., Virág L., Gergely P., Leibovich S.J., Pacher P., Sun C.-X.X., Blackburn M.R., Vizi E.S., Deitch E.A. (2007). A2A adenosine receptors and C/EBP?? are crucially required for IL-10 production by macrophages exposed to Escherichia coli. Blood.

[61] Ruffell D., Mourkioti F., Gambardella A., Kirstetter P., Lopez R.G., Rosenthal N., Nerlov C. (2009). A CREB-C/EBPbeta cascade induces M2 macrophage-specific gene expression and promotes muscle injury repair. Proc. Natl. Acad. Sci. USA.

[62] Natsuka S., Akira S., Nishio Y., Hashimoto S., Sugita T., Isshiki H., Kishimoto T. (1992). Macrophage differentiation-specific expression of NF-IL6, a transcription factor for interleukin-6. Blood.

[63] Hu B., Ullenbruch M.R., Jin H., Gharaee-Kermani M., Phan S.H. (2007). An essential role for CCAAT/enhancer binding protein beta in bleomycin-induced pulmonary fibrosis. J. Pathol..

[64] Hu B., Wu Z., Jin H., Hashimoto N., Liu T., Phan S.H. (2004). CCAAT/enhancer-binding protein beta isoforms and the regulation of alpha-smooth muscle actin gene expression by IL-1 beta. J. Immunol..

[65] Benjamini Y., Hochberg Y. (1995). Controlling the False Discovery Rate: A Practical and Powerful Approach to Multiple Testing. J. R. Stat. Soc. Ser. B.

